# Evaluating the Impact of Faculty Development Programs in Generating Self-Efficacy and Competency Among Medical Teachers in India

**DOI:** 10.7759/cureus.65150

**Published:** 2024-07-22

**Authors:** Bishwajeet Saikia, Sudipta D Baruah, Chau Pingsaymang Manpoong, Amitav Sarma, Mohan K Ram, Sarah Ralte, Priyanka Barua, Ofisha Mary Kurbah, Somjita Datta, Atula Imchen, Irom Sapana

**Affiliations:** 1 Anatomy, North Eastern Indira Gandhi Regional Institute of Health and Medical Sciences (NEIGRIHMS), Shillong, IND; 2 Data Labelling, KaliberAI, Guwahati, IND; 3 Anatomy, Teerthanker Mahaveer Medical College, Moradabad, IND; 4 School of Social Work, Martin Luther Christian University, Shillong, IND

**Keywords:** self-efficacy, competency-based medical education, competency, faculty development program, medical education

## Abstract

Background

Maintaining the quality of teaching across India is a challenge. Teachers are equally responsible for patient care and administration. The importance of training medical teachers under the various faculty development programs (FDPs) is well accepted. A mechanism to evaluate the competencies acquired by medical teachers after attending FDPs becomes equally important. In the present study, we evaluate the impact of the various FDPs on medical teachers.

Methodology

A cross-sectional study was conducted for one year among 50 medical teachers attending FDPs. Ethical clearance was taken from the Institutional Ethical Committee. For quantitative data collection, the questionnaire was validated by the Scientific Approval Committee of the Institute. The study questionnaire was filled in by the participants just before and three months after attending FDPs. For qualitative data collection, in-depth Interviews (IDIs) using the Kirkpatrick model of learning evaluation were conducted. Descriptive statistics were presented as frequencies and percentages. The thematic areas of self-efficacy and teaching competency before and after FDPs were tested using the chi-square test. P-values <0.05 were considered significant.

Results

There was a significant increase in self-efficacy (300 vs. 426, p* *= <0.0001) and teaching competency (456 vs. 608, p = <0.0001) in the domains of teaching difficult students and motivating students for innovative projects. Improvement in communication skills and ability to engage the students were noteworthy in teaching competency. IDIs revealed that FDPs are essential for the efficient delivery of the competency-based medical curriculum.

Conclusions

FDPs play a key role in bringing about significant improvement in generating self-efficacy and teaching competencies among medical teachers. FDPs may be incorporated into the postgraduate medical curriculum itself.

## Introduction

The Indian medical education system produces one of the highest numbers of doctors globally and most of them are involved in medical teaching [1,2]. Taking into account the burden of clinical care, the administrative work with the existing shortage of doctors maintaining the quality of education becomes a challenge in the country. Measures such as the introduction of innovative teaching programs like competency-based medical education (CBME) are being taken up by the Medical Council of India (MCI), now the National Medical Commission (NMC), to improve the quality of medical education [3]. Subsequently, faculty development programs (FDPs) such as the Basic/Revised Basic course/Curriculum Implementation Support Program were introduced by the MCI long back to train medical teachers (MTs) [1]. These programs are expected to prepare MTs for the newly laid CBME as well as to bring about a positive impact on the overall teaching quality [4].

While these FDPs conducted at regular intervals at various medical teaching Institutes become crucial to training MTs, a mechanism to evaluate this becomes equally important to ensure their impact on the upliftment of overall teaching quality. Further, the deficiencies detected through these evaluations can be acted upon to revisit and revise the existing approach [5]. Therefore, in this project, we aim to study the impact of FDPs among MTs in generating self-efficacy and teaching competency.

## Materials and methods

Study design

A cross-sectional study with both qualitative (in-depth interviews (IDIs) with MTs) and quantitative (questionnaire-based) approaches was used.

FDP interventions

The Basic/Revises Basic course workshops were designed following the NMC guidelines by the Medical Education Unit at our institute. The FDPs also included non-NMC workshops specially designed for senior residents. Trainers for these workshops were the faculties trained from NMC-designated centers across India. The framework for these sessions included the development of CBME and the professional growth of teachers.

Study participants and duration

The study was conducted for one year. The participants included 50 medical teachers (faculties and senior residents) who attended the FDPs (Basic/Revised Basic course workshops).

Inclusion and exclusion criteria

MTs who were not trained/exposed to FDPs and gave prior consent for the study were included, whereas MTs who were already trained/exposed to FDPs and who refused to give consent for the study were excluded.

Ethical considerations

The study was approved by the Institutional Ethical Committee, North Eastern Indira Gandhi Regional Institute of Health and Medical Sciences (approval number: NEIGR/IEC/M14/F1/2021). Informed consent was taken from the participants before conducting the study. The collected data before and after FDPs as well as feedback from the participants after the FDPs were kept anonymous.

Data collection

Quantitative

The questionnaire was validated by the Scientific Approval Committee of the institute. The study questionnaire was filled up by the participants just before and three months after attending FDPs. The faculty self-efficacy test was a self-reporting, four-point Likert scale based on thematic areas such as skill development, workplace-based social interactions, job accomplishment, and coping with job stress [6]. Assessment of teaching competency was done using a self-reporting Likert scale based on domains such as communication skills, understanding and organizing the subject, planning learning experiences, engaging and supporting all students, attending to the students’ queries, accessing students’ learning, and receiving unbiased feedback from the students.

Qualitative

IDIs using the Kirkpatrick model of learning evaluation, (reaction, learning, impact, and results) were conducted for MTs who underwent FDPs at our institute [7]. For the IDIs, the participants were contacted initially during the FDP sessions. After obtaining passive consent at the initial contact, those who agreed were interviewed three months post-FDPs at a predetermined time convenient to them. An interview guide was written before the interview which was based on the study objective. However, extracts from suggestions, comments, and reflections during the interview were taken wherever felt relevant to the study. Handwritten notes were taken to keep the interview record.

Data analysis

Descriptive statistics were presented as frequencies and percentages. The thematic areas of self-efficacy and teaching competency before and after FDPs were tested using the chi-square test. The attitude statements that were scored as “not at all effective” and “barely effective” were presented as “not effective” and those scored as “moderately effective” and “exactly effective” were presented as “effective.” P-values <0.05 were considered significant.

## Results

Quantitative component

**Figure 1:**
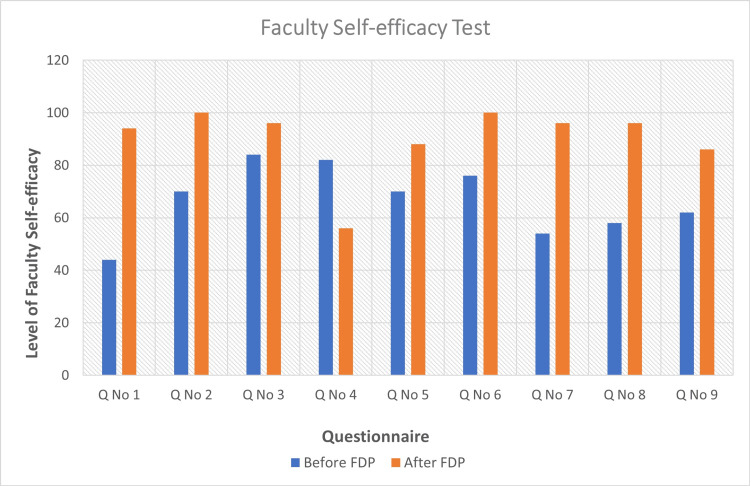
Faculty self-efficacy test. Bar diagram representing the assessment of teacher self-efficacy before and after participation in FDPs as per the question numbers provided in Table 1. FDP = faculty development program

**Figure 2:**
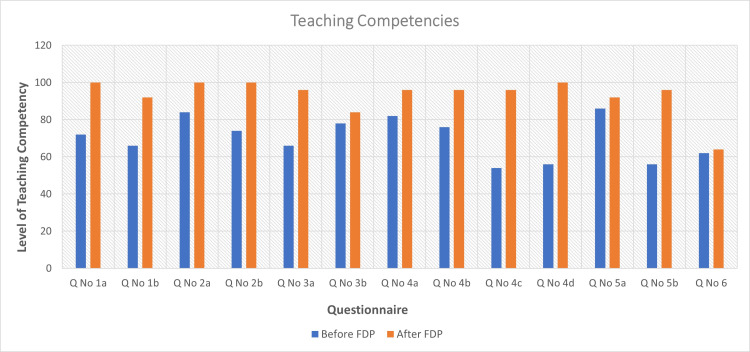
Teaching competencies. Bar diagram representing the assessment of teaching competency before and after participation in FDPs as per the question numbers provided in Table 2. FDP = faculty development program

A total of 50 MTs underwent at least a single FDP session from 2020 to 2021, of whom 37 (74%) were faculty members, and 14 (26%) were senior resident doctors. The median year of teaching experience was one year. The mean score of teacher self-efficacy increased from 300 out of 450 to 426 (p < 0.0001) (Table 1, Figure 1). There were significant improvements in all domains. Remarkable improvements were observed in the thematic areas of self-efficacy in the domains of teaching difficult students (p < 0.0001), communicating with difficult students (p < 0.0001), and motivating students for innovative projects (p < 0.0001). The mean score of teaching competency increased from 456 out of 650 to 608 (p < 0.0001) (Table 2, Figure 2). There was a remarkable improvement in communication skills in the classrooms (p < 0.0001). Further, domains such as self-directed reflective learning in students and promoting individual as well as group responsibility were significantly improved (p < 0.0001).

**Table 1:** Assessment of teacher self-efficacy before and after participation in FDPs. FDP = faculty development program

	Faculty self-efficacy test					
Question number		Before FDP		After FDP		Chi-square
		Not effective (%)	Effective (%)	Not effective (%)	Effective (%)	P-value
1	Teaching relevant content to the most difficult students	56	44	6	94	<0.0001
2	Communicating with difficult students	30	70	0	100	<0.0001
3	Efficiency in addressing students’ needs	16	84	4	96	0.0956
4	Maintaining composure while teaching even when disturbed	18	82	4	56	0.0552
5	Responsiveness to students’ needs even on a bad day	30	70	12	88	0.0495
6	Trying to exert a positive influence on the personal and academic development of students	24	76	0	100	0.0007
7	Coping with the systemic constraints and continuing to teach well	46	54	4	96	0.001
8	Motivating students to participate in innovative projects	42	58	4	96	<0.0001
9	Carrying out innovative projects even when opposed by skeptical colleagues	38	62	14	86	0.021
	Total	33.33	66. 67	5.33	94. 67	<0.0001

**Table 2:** Assessment of teaching competency before and after participation in FDPs. FDP = faculty development program

	Teaching competencies					
Question number		Before FDP		After FDP		Chi-square
		Not effective (%)	Effective (%)	Not effective (%)	Effective (%)	P-value
1a	I am confident that I can communicate very well with all my students during classroom sessions as well as outside	28	72	0	100	<0.0001
1b	I am communicating effectively with the students to bring about a behavioral change	34	66	8	92	0.0032
2a	I know that I always ensure to understand and organize the subject before	16	84	0	100	0.0099
2b	I always try to connect students’ prior knowledge to the learning goals	26	74	0	100	0.0004
3a	While planning the lecture, I am instrumental in choosing the best learning experiences for students	34	66	4	96	0.0004
3b	I always ensure to use resources, materials, and technologies to make the subject matter more accessible	22	78	16	84	0.6102
4a	I am confident that I always support my students and that I am always available for communication	18	82	4	96	0.0552
4b	I always try to engage students in problem-solving and critical thinking	24	76	4	96	0.0095
4c	I always promote self-directed reflective learning in students	46	54	4	96	<0.0001
4d	Promoting individual as well as group responsibility is a part of my regular teaching	44	56	0	100	<0.0001
5a	I always try hard to attend to student queries while I access their learning	14	86	8	92	0.5227
5b	I communicate with students on their progress of learning	44	56	4	96	<0.0001
6	Receiving unbiased feedback has been always a part of my lesson plan	38	62	32	64	0.675
	Total	29.85	70.15	6.46	93.54	<0.0001

Qualitative component

The IDIs explored the views of MTs toward the relevance of the FDPs, the approach of the training program, and its impact on teaching undergraduate medical students. The IDIs were conducted around the thematic areas of teaching self-efficacy and teaching competency.

Importance of FDPs

There was an overall realization from the participants about the significance of getting trained under the various FDPs which changed their approach toward teaching undergraduate students. The new medical curriculum identified the competencies in different learning domains. It was not very surprising to find that some of these terminologies such as competency and learning domains were very new to some of the participants, especially the senior resident doctors. Even though many of them were aware of these terminologies and were using them during their classes, some were not very sure about terminologies. “We hardly came across these terms in our Post Graduate curriculum, where we are still used to the traditional teaching, only during the FDP sessions we understood these terms” - P1. The participants felt very strongly about the introduction of these training programs in the postgraduate curriculum as the new CBME has been already introduced in undergraduate medical education. Most of them agreed that the duration and frequency of such FDP sessions should be increased. “These types of sessions should be happening more frequently and can be made short and targeted towards small topics” - P2. 

Understanding the approach toward addressing all learning domains while teaching the undergraduates, especially the affective domain, was something the participants learned during this training. “Earlier, the affective domain was something which was learned from observing our seniors. Sometimes that can be very subjective and was never approached in such a structured manner” - P3. “The training helped us in understanding and revisiting our attitude towards the patient in the ward as well the students during the teaching-learning session” - P3.

Quality and professional improvement

The participants felt that FDPs helped them a lot in understanding the new CBME curriculum and they could organize the teaching-learning interactions more efficiently and be more aware of their roles during each teaching session after attending the FDPs. The sessions helped them to systematically approach the curriculum and teaching. The concept of problem-based and self-directed learning was better understood. Group dynamics and their role in adult learning were better appreciated. “An overall knowledge base was gained on “how to teach” and perhaps if not for the online classes during the COVID-19 pandemic, we could have implemented the ideas in a more effective way during the offline classes” - P4. “Understanding and organizing the subject before a class and choosing the best learning experience for them is what I am trying to do in my online classes now” - P5.

Time constraints while implementing the concepts of FDPs during a class was a factor that was mentioned by most participants. “Conducting effective sessions for the students takes time, especially when the whole concept is new for us. But I feel once we are used to it, it will be a lot easier” - P5. The participants also emphasized that after the FDPs they feel that they can communicate better with the students as well as with the patients.

Challenges in implementing CBME

The sessions for the basic and revised basic workshops in medical education were by the trainers who were trained in NMC-designated centers across the country. Most participants were satisfied with the quality of training and opined that the institute should conduct more training in the future and at least attempt to conduct some sensitization programs for the postgraduates.

“The competency booklet of the CBME was a concept which was very new to me, most of us never heard of this earlier. Definitely, such sessions are helpful and should be carried out continuously, especially during our post-graduate training” - P7.

Even though the overall impact of the FDPs was felt to be positive by the participants, there were comments concerning time constraints and lack of faculty strength which might affect the long-term sustainability of the FDPs.

## Discussion

FDPs represent a crucial initiative aimed at enhancing efficiency within medical education. Recent years have seen diverse approaches to delivering FDPs worldwide. With its vast number of medical colleges and, consequently, a higher count of medical teachers, India holds a prominent position globally [8]. However, there is an urgent necessity to adopt a systematic approach to faculty development to ensure quality education in alignment with the 21st-century health challenges, as outlined by the MCI, now the NMC [9]. In India, FDPs are primarily organized by medical colleges and universities through Basic, Revised Basic, and Advanced Courses on pedagogy. These courses aim to improve the quality of medical education by equipping MTs with the necessary knowledge and skills. Basic courses in Medical Education focus on imparting fundamental knowledge, skills, and attitude changes across various teaching and assessment domains [1]. FDPs play a central role in delivering medical education that is adaptable to changes in healthcare systems, societal expectations, and the shift toward workplace-based learning approaches. In India, FDPs began in 1976, evolving through initiatives such as the contribution of National Teacher Training Centres, medical education units, the Consortium of Medical Institutions for Reform of Medical Education, and the Foundation for Advancement in International Medical Education and Research, Philadelphia [10]. While the NMC Basic course has become compulsory for MTs up to the professor level, it is also recommended that medical education units in all medical colleges train existing MTs in the revised Basic course and conduct the course for newly appointed faculty twice a year [10]. Even though there are norms under which pre- and post-test are used to assess the quality of these workshops, evaluating MTs in acquiring these competencies in the workplace becomes crucial. Consequently, the number of trainings conducted and the number of participants trained cannot be the only parameter for determining the teaching skills [4]. Programs should be designed to educate policymakers about recent advances in medical education worldwide, focusing on moral purposes, capacity building for change, understanding the change process, fostering a learning culture, and promoting development at all levels. Tenzin et al. observed significant enhancements in self-efficacy and teaching abilities among postgraduate faculty members following their participation in FDP interventions [11]. In most countries, there is evidence that educators of health professionals are insufficiently prepared as teachers and trainers, even though their clinical knowledge and skills may be good [1]. Where elementary, primary, and secondary school teachers must undergo training in formal schools or colleges of education to be eligible for appointment and promotion, there is no such requirement for appointment of teachers in medical colleges in India [1]. In our study, most participants commented that FDP interventions should start as early as in the postgraduate training so that future MTs are well acquainted with the concepts of medical education and can evolve as competent and effective teachers. Additionally, it was noted that these FDPs contributed to the early professionalization of postgraduate medical education in a medical university in Bhutan [11]. However, in India, there are no structured FDP mandates by the NMC to train senior residents, and FDPs for senior residents are usually initiatives from the individual Institution. Here, it must be emphasized that with the shortage of MTs in the current medical education system, senior residents become an important component of efficient medical education despite that NMC-initiated FDPs do not include senior residents [12]. Training residents would increase their awareness of faculty and program expectations, which would improve their ability to be effective learners, increase faculty and resident alignment, and reduce the likelihood of conflict and confusion. Residents reported positive changes and self-confidence in attitudes toward teaching [12,13]. In a study conducted by Hakim et al., to evaluate the efficacy of adapted Medical Education Technology (MET) workshops for resident doctors resulted in positive outcomes. MET workshop was an effective tool for improving knowledge of medical education as well as changing the perception of a teacher among resident doctors [14]. Similar to an FDP, a resident development program might be a new horizon that should be discussed to increase the applicability of the article in the medical education field. Our study which also included senior residents found improvement in self-efficacy and teaching competencies of MTs who underwent FDPs in India which were statistically significant. Expectations of self-efficacy determine whether instrumental actions will be initiated, how much effort will be expended, and how long it will be sustained in the face of obstacles and failures. According to theory and research, self-efficacy makes a difference in how people think, feel, and act. A strong sense of competence facilitates cognitive processes and performance in a variety of settings, including quality of decision-making and academic achievement [15]. Our study found a significant improvement in the domains of self-efficacy in MTs after attending the FDPs. In India, FDPs have been known to have a positive impact on personal/professional/institutional growth [2].

FDPs have not only benefitted resident learning and improvement in service delivery at the hospital, but it has also brought improvement in the working environment among faculty members through better communication skills. An increased proportion of faculty members reported the positive impact of FDPs on their communication skills concerning the delivery of subject knowledge and effective communication to bring about behavioral and attitudinal change in their students. FDPs brought examples of the benefits of better communication skills for both residency training and patient care. Even though the FDP showed some positive results, it is necessary to mention the limitations. As the study was conducted in one teaching medical institute in North-East India, it might not represent the exact scenario of FDPs across India but it highlights their inevitability. A bigger sample size would reflect better evidence.

## Conclusions

FDPs brought about improvements in self-efficacy and teaching competencies among MTs, which were statistically significant. More MTs need to be trained in FDPs for the overall growth of the medical education system. It is suggested that FDPs may be introduced early as a part of the postgraduate curriculum.

## Appendices

The questionnaire was validated by the Scientific Approval Committee of the Institute. The study questionnaire was filled up by the participants just before and three months after attending the FDPs.  Assessments were done using a self-reporting, four-point Likert scale.

**Table 3:** Faculty self-efficacy test questionnaire. Response format: (1) not at all true, (2) barely true, (3) moderately true, (4) exactly true. rBCW = revise Basic course workshop

Faculty self-efficacy test		Before rBCW
1.	I am convinced that I am efficient in successfully teaching all relevant subject content to even the most difficult students	
2.	When I try really hard, I am able to reach even the most difficult students	
3.	I am convinced that, with time, I will be more efficient in helping address my students’ needs	
4.	Even if I am disrupted while teaching, I am confident that I can maintain my composure and continue to teach well	
5.	Even if I am having a bad day, I am confident in my ability to be responsive to my students’ needs	
6	If I try hard enough, I know that I can exert a positive influence on both the personal and academic development of my students	
7.	I am convinced that I can invent ways to cope with system constraints (such as budget cuts and other administrative problems) and continue to teach well	
8.	I know that I can motivate my students to participate in innovative projects	
9.	I know that I can carry out innovative projects, even when I am opposed by skeptical colleagues	

**Table 4:** Assessment of teaching competencies. Response format: (1) not at all true, (2) barely true, (3) moderately true, (4) exactly true. rBCW = revise Basic course workshop

Assessment of teaching competencies			Before rBCW
1.	Communication skills	I am confident that I can communicate very well with all my students during classroom sessions as well as outside	
		I am communicating effectively with the students to bring about a behavioral change	
2.	Understanding and organizing the subject	I know that I always ensure to understand and organize the subject before	
		I always try to connect students’ prior knowledge to the learning goals	
3.	Planning learning experiences	While planning the lecture, I am instrumental in choosing the best learning experiences for students	
		I always ensure to use resources, materials, and technologies to make the subject matter more accessible	
4.	Engaging and supporting all students	I am confident that I always support my students and that I am always available for communication	
		I always try to engage students in problem-solving and critical thinking	
		I always promote self-directed reflective learning in students	
		Promoting individual as well as group responsibility is a part of my regular teaching	
5.	Attending students’ queries and accessing learning	I always try hard to attend to students’ queries while I access their learning	
		I communicate with students on their progress of learning	
6	Receiving feedback from the student	Receiving unbiased feedback has been always a part of my lesson plan	
